# Bridging the Gap in Adult Dyslexia Research: Assessing the Efficacy of a Linguistic Intervention on Literacy Skills

**DOI:** 10.1007/s11881-024-00314-x

**Published:** 2024-09-23

**Authors:** Maria Vender, Denis Delfitto

**Affiliations:** Department of Cultures and Civilizations, University of Verona, Viale Dell’Università 4, 37129 Verona, Italy

**Keywords:** Developmental Dyslexia, Dyslexic adults, Literacy intervention, Adult literacy, Linguistic profile in dyslexia

## Abstract

While developmental dyslexia has been extensively studied in children, research on adults is still rather limited. This paper aims to bridge the gap in existing research by presenting the findings of a study that examined the reading and spelling skills of adults with dyslexia and assessed the effectiveness of a linguistic intervention designed to improve their literacy abilities. To address this issue, we first compared the profiles of 44 adults with dyslexia (age range: 16–30 y.o.) and 44 age-matched typical readers across tasks assessing reading, spelling, phonological awareness, morphological awareness and lexical access in Italian. The findings underscored pervasive impairments in dyslexia across all measured dimensions, reaffirming the persistent nature of language and literacy challenges into adulthood. In pursuit of the second objective, the study explored the potential for literacy skill improvement in adults with dyslexia through the implementation of a specialized intervention proposed to 24 dyslexic adults and delivered via a web application. The intervention program yielded positive outcomes in the experimental group, demonstrating significant improvements in word and text reading, spelling, and speed of phonological elaboration. This study, hence, contributes not only to our understanding of developmental dyslexia in adulthood but also emphasizes the tangible benefits of targeted linguistic interventions, thereby offering practical implications for the amelioration of literacy skills in this population.

## Introduction

### Developmental Dyslexia: reading development, linguistic predictors and intervention in children

Developmental dyslexia (dyslexia henceforth) is a neurodevelopmental disorder that interferes with the acquisition of literacy skills and cannot be attributable to low intelligence level, neurological deficits or poor educational opportunities (First, 2013). It is a genetic and inheritable condition whose prevalence is estimated approximately 7% (Yang et al., 2022), twice as prevalent among boys compared to girls, with differences observable across countries, depending on the opacity of the orthographic system considered.

While the most apparent symptoms of dyslexia manifest in reading difficulties, affecting accuracy, fluency, and comprehension, as well as spelling errors, dyslexia is also characterized by marked deficits affecting different levels of linguistic competence. In particular, difficulties are reported in phonological skills, in the identification and manipulation of phonemes (Bishop & Snowling, 2004; Ramus & Szenkovits, 2008; Vellutino et al., 2004; Vender & Melloni, 2021), morphological abilities, especially in the application of morphological rules to invented words (Casalis et al., 2004; Joanisse et al., 2000; Melloni & Vender, 2022), grammatical competence, in the comprehension and production of morphosyntactically complex structures (Arosio et al., 2017; Bar-Shalom et al., 1993; Cardinaletti et al., 2022) and lexical access, assessed by means of rapid naming tasks (Denckla & Rudel, 1976; Nicolson & Fawcett, 1994) and pragmatic competence (Cappelli et al., 2022). Working memory (WM), executive functions and automatization of skills are also substantially compromised, detrimentally affecting performance in tasks that demand significant cognitive resources or in dual-tasks (Nicolson & Fawcett, 2008; Varvara et al., 2014; Vender, 2017).^1^

While the debate on the causes of dyslexia is still open and various theoretical frameworks have been developed to account for its manifestations, there is ample consensus on the role of phonological awareness, which is fundamental for literacy acquisition and in particular for learning and automatizing grapheme-phoneme conversion rules (Snowling et al., 2020). Phonological skills are indeed determinant to enable individuals to learn and establish associations between the orthographic and phonological representations of words.

According to the dual-route model (Coltheart et al., 2001), two distinct pathways can be used for reading, based on the nature of the written stimuli and on the reader’s proficiency and age. In the sublexical route, which is typically used to read unfamiliar or infrequent words and nonwords, the stimulus is decomposed into its minimal components, which are then associated with their phonological representation applying conversion rules. The lexical route is instead used to decode familiar words that are already stored in the reader's mental lexicon, so that the reader accesses the whole-word representation directly from memory, which allows for rapid and automatic recognition. This route is typically used by more proficient readers to decode familiar words or words that have irregular spellings, thus bypassing phonological decoding. Expert readers can flexibly use both routes depending on the familiarity and complexity of the word to be read; moreover, as their proficiency increases, the connections between phonological and orthographic forms become increasingly stronger and are automatized, leading to more rapid and effective decoding (Ehri, 2005).

Acquiring literacy is heavily influenced by the characteristics of the orthographic systems, in terms of both granularity and transparency (Wydell & Butterworth, 1999). In alphabetic writing systems, mappings are fine-grained since they are established at the level of single phonemes. However, these mappings are much more consistent in transparent orthographies, where there are mainly regular, one-to-one correspondences between phonemes and graphemes (as in Italian and German), compared to opaque orthographies (as English and French), which are instead characterized by many-to-many correspondences between sounds and letters. Orthographic depth then significantly influences learning to read, which is notably easier in transparent orthographies, and it also impacts the manifestations of reading deficits in dyslexia: difficulties in decoding, especially in accuracy, are indeed markedly higher in English than in German readers with dyslexia (Landerl et al., 2013; Ziegler & Goswami, 2005). Nevertheless, independently from the characteristics of the language considered and of their writing system, phonological awareness constitutes a universal, strong predictor of the development of reading skills, and is consistently impaired in individuals with dyslexia, in both children and adults (see Carioti et al., 2021 for a recent meta-analysis considering different age groups and orthographies). In addition, lexical access, as measured by Rapid Automatized Naming (RAN), is another significant cognitive marker and predictor of dyslexia across ages and orthographies (Carioti et al., 2021). RAN is indeed critical for reading as it requires the rapid retrieval and articulation of phonological information associated with visual symbols, an ability that is essential for efficient phonological processing, and for achieving fluent reading.

More recently, it has been observed that morphological awareness is another relevant predictor of reading skills in dyslexia, becoming increasingly important as the reader’s proficiency increases, even surpassing the role of phonology in older children (Giazitzidou & Padeliadu, 2022; Rothou & Padeliadu, 2019; Torppa et al., 2010). The contribution of morphology in decoding is crucial as it supports the identification of bases and affixes in complex words, as evidenced by the fact that morphologically complex words are typically read faster than simple words matched for length and frequency by both typical and atypical readers, independently from age (Burani et al., 2008; Carlisle & Stone, 2005; Levesque & Deacon, 2022).

Given their fundamental contribution to reading development, both phonological and morphological competence have an important role in treatments for dyslexia. It is indeed essential to remark that an early intervention can significantly alleviate reading and spelling difficulties. A growing body of research has demonstrated the effectiveness of specialized training programs in enhancing literacy skills and giving dyslexics the opportunity to overcome their reading and spelling challenges, thus paving the way for a more positive and fulfilling educational experience (Snowling et al., 2020). As reported by previous meta-analyses, an early reading intervention proposed to children can have robust effects, significantly reducing their difficulties (Gersten et al., 2020; Neitzel et al., 2022). Particularly effective are those interventions that combine explicit and systematic instruction in letter-sound relationships and sound blending, such as phonics instruction, with reading fluency training and phonemic awareness training (Galuschka et al., 2014; see McArthur et al., 2018 for a systematic review of the phonics treatment). Another valuable strategy for fostering reading proficiency consists in developing the participants’ morphological awareness: by promoting a morphological analysis of complex words through the identification of bases and affixes, it is possible to strengthen both decoding and comprehension (Bowers et al., 2010; Levesque & Deacon, 2022). An alternative approach aiming to automatize and speed up decoding is the Reading Acceleration Paradigm, which involves constrained reading exercises where students are compelled to read at a faster pace than their usual reading rate, guided by computerized training programs (Breznitz, 1997, 2001; Breznitz & Share, 1992). It has been shown that this type of training can improve fluency among children, without compromising comprehension (Irausquin et al., 2005), and regardless of the orthographic complexity of the writing system (Horowitz-Kraus et al., 2014).

Again, literacy interventions have traditionally focused on children and adolescents, with relatively little attention given to the importance of literacy training for adults. Therefore, research on dyslexia in adults remains a vast and unexplored territory, with much to be gained in understanding their linguistic and cognitive profiles, the evolution of the disorder across the lifespan, and the development of effective interventions.

### Dyslexia in adults: challenges and intervention strategies

While access to written resources is increasingly crucial in modern society, extending far beyond formal education, the needs of adults with dyslexia have so far received relatively scarce attention. If it is certainly essential to comprehensively study the linguistic and cognitive profile of children with dyslexia to develop always more appropriate identification methods and timely intervention strategies, focusing only on children does not permit us to understand how difficulties evolve over time. It must indeed be remembered that dyslexia is a condition that persists across the lifespan and that, although its primary manifestations are mostly evident at school, it can have repercussions that extend beyond educational settings. At the professional level, indeed, people with dyslexia tend to show lower employment rates, reduced earnings, placement in lower-skilled positions, and diminished job satisfaction (McLoughlin & Leather, 2013; Nalavany et al., 2018).

The studies conducted so far on adults with dyslexia indicate that, despite years of accumulated reading experience, literacy difficulties remain marked among adults: the studies reviewed in the meta-analysis by Swanson and Hsieh (2009) reported the persistence of difficulties in word recognition, pseudoword reading, reading comprehension, and spelling, with no effects of age or gender. A later meta-analysis by Swanson (2012) confirmed the presence of significant deficits in measures of vocabulary, math, spelling, naming speed, phonological processing, and verbal memory, indicating that these deficits are persistent across age and independent of gender, ethnicity, and socioeconomic status. As shown by a more recent meta-analysis (Reis et al., 2020), literacy difficulties in adults with dyslexia tend to be more pronounced in reading speed than in accuracy; fluency emerges indeed as a significant challenge across orthographies, characterized by slow and effortful decoding, while inaccurate decoding is more prevalent in opaque orthographies. Similarly, spelling remains a core deficit for adults with dyslexia, but difficulties tend to be less pronounced in transparent orthographies. Reading comprehension, instead, is reported as impaired across writing systems, albeit being less conspicuous than reading and spelling deficits.

Beyond literacy difficulties, adults continue to show deficits in the linguistic domain, especially in phonological competence (Bruck, 1992) and lexical access measured with rapid naming (Vukovic et al., 2004). Verbal memory deficits are also observed and expected to hinder efficient access to the phonological and executive resources required for reading and writing processes (Reis et al., 2020). As found in children, both phonological awareness and rapid naming are reliable predictors of reading difficulties (Swanson & Hsieh, 2009). However, while phonological awareness emerges as a stronger predictor of reading difficulties in opaque orthographies, rapid naming remains a robust predictor across all writing systems (Reis et al., 2020).

Importantly, most studies have focused on English, which has an opaque orthography, which raises concerns about the generalizability of these findings to languages with different writing systems (see also Carioti et al., 2021). Reis et al. (2020) made a valuable contribution by considering the role of orthographic transparency, classifying the studies reviewed in three categories based on orthographic transparency. Anyway, studies on opaque orthographies remained the majority (67.4%), while studies with intermediate or low opaqueness were more limited (respectively 19.7% and 12.9%), suggesting the need for research on different languages, especially with transparent orthographic systems.

An appropriate treatment can in fact open doors to higher education and reduce dropout rates, which are typically higher among dyslexic individuals compared to typical readers (Davis et al., 2008; Moriña, 2017). In line with this, the recent growing awareness of learning disabilities and the increased sensitivity towards these issues encouraged more individuals with dyslexia to pursue higher education, leading to higher enrollment rates and more positive outcomes (Richardson, 2021; Shaywitz et al., 2020).

It is moreover important to remark that literacy skills can be enhanced also in adulthood. Although research on literacy interventions in adults is still rather limited, the few studies conducted so far suggest that both reading and spelling can be enhanced in adulthood as well (see Vender et al. (2022) for a recent systematic review of the literature). As in children, interventions proposed to adults are typically based on training phonological skills, as well as on automatizing decoding and speeding up phonological processing, due to their crucial role in reading development. More particularly, phonics interventions, generally proposed with a multisensory instruction and thus with a simultaneous engagement of multiple senses (auditory, visual, kinesthetic-tactile), are reported as effective (Eden et al., 2004; Greenberg et al., 2011; Guyer & Sabatino, 1989; Sabatini et al., 2011). Incorporating morphological awareness into reading interventions can yield significant benefits for adults with dyslexia too, improving word recognition as well as promoting a deeper understanding of word structure (Bar-Kochva, 2016; Gray et al., 2018). Computerized interventions using the reading acceleration paradigm (Breznitz et al., 2013; Horowitz-Kraus, 2016) or addressing working memory (Shiran & Breznitz, 2011) were effective too. Significant gains are reported in word and nonword reading as well as in passage reading; only very few studies instead found the presence of improvements in reading comprehension (Sabatini et al., 2011; Shiran & Breznitz, 2011). Effects on spelling were only rarely considered and reported (see Bar-Kochva, 2016 for an exception). Improvements in phonological competence were indeed observed by Eden et al. (2004) and Shiran and Breznitz (2011). Notably, there have been reports of neurological changes alongside with behavioral measures, indicating that the brain's plasticity remains significantly high also in adulthood (Eden et al., 2004; Horowitz-Kraus, 2016; Shiran & Breznitz, 2011). Also in this case, studies are mostly conducted on English participants (see Vender et al., 2020 for more details about the studies conducted).

## The current study

Given the aforementioned considerations, our study aimed to provide new insights into the literacy and linguistic profile of adults with dyslexia in Italian, a language with a highly transparent orthographic system that has received little consideration so far. This study had, therefore, a three-fold objective. First, we aimed to provide a comprehensive assessment of the participants’ literacy skills, to verify whether difficulties in reading and spelling persist in adulthood as well. Secondly, we aimed to examine in detail the linguistic profile of adults with dyslexia, focusing particularly on those skills that are known to predict reading proficiency, as discussed above, i.e., phonological skills, morphological abilities, and lexical access, in order to verify whether difficulties in these domains are still pronounced in adults with dyslexia. Based on these results, we then developed an original linguistic intervention designed to enhance their literacy skills. Our last research aim was to assess the effectiveness of this intervention, thus ascertaining whether reading skills can be successfully trained also in adults with dyslexia.

## Method

### Participants

The experimental protocol was administered to 88 participants, divided into 2 groups: 44 young adults with dyslexia (age range: 16–30, mean age: 20.59 y.o., SD = 3.33) and 44 age-matched typical readers (age range: 16–30, mean age: 22.47 y.o., SD = 3.55). We decided to include also 16 and 17 years old participants because intervention studies often involve younger participants, up to 13–14 years old, and late adolescents are typically not considered (McArthur et al., 2018), whereas they might be included in reading programs for adults (see Greenberg et al., 2011; Guyer & Sabatino, 1989; Sabatini et al., 2011). Moreover, late adolescents tend to perform more similarly to adults than to children in literacy and language tasks (see also Wiseheart et al., 2009 for a study on young adults with dyslexia comprising also 16-year-old participants).

All dyslexics had been independently diagnosed based on standard criteria (ICD-10; WHO, 2004) and they had no diagnosed or reported oral language problems or other physical, cognitive or neurological deficits. Typical readers had no diagnosed or referred cognitive deficits, no reading, learning or language disorders. Most participants were high-school or university students (38 dyslexics, 32 controls) while the others (6 dyslexics and 12 controls) were already employed. All participants were born in Italy, lived in the same area in the North-East of Italy and were native speakers of Italian.

To ensure comparability amongst the two groups, we administered the Raven Standard Progressive Matrices (SPM) task (Raven, 2008). No participants scored lower than 2 SDs below the mean; we fitted a linear model to predict performance in the SPM with Group (controls: M = 0.65, SD = 0.62; dyslexics: M = 0.34, SD = 1.05) and no statistically significant differences were observed (β = -0.32, t(86) = -1.73, p = 0.09).

All participants were administered a set of tasks aimed at assessing their literacy and linguistic skills (see Sect. "Materials"). Following this pre-intervention assessment, dyslexics were randomly split into an experimental group, which was administered the literacy intervention outlined in Sect. "Literacy intervention", and in a control group, which did not receive any specific intervention. Both groups were then retested to evaluate the training’s effectiveness, as will be explained below.

### Materials

#### Literacy measures

To assess the literacy skills of the participants, we administered a set of standardized tasks used for the evaluation of the reading and spelling skills of young Italian adults. More particularly, we evaluated text reading, word reading and nonword reading, using the LSC-SUA battery (Montesano et al., 2020) and asking subjects to read aloud respectively a short text, four series of isolated words varying in frequency and concreteness (e.g. *ombra*, ‘shadow’: high frequency and concreteness; *frugalità,* ‘frugality’: low frequency and concreteness) and two lists of isolated nonwords varying in length and complexity (e.g. *mapri*: low length and complexity; *mendantofo*: high length and complexity). Both accuracy and reading time were measured and transformed in z-scores. A composite score encompassing accuracy and speed in text, word, and nonword reading was then computed, assigning equal weight to the participant's z-scores in decoding accuracy and speed. In specific terms, the composite score was determined by combining 0.5 times the z-scores in reading accuracy with 0.5 times the z-scores in reading speed.

We also administered a more complex measure, a lexical decision task under articulatory suppression (LCS-SUA battery), which has been found very reliable for discriminating dyslexic adults from normal readers (Re et al., 2011). In this task participants were presented with a list of 24 words (e.g. *importanza*, ‘importance’) and 24 non-words (e.g. *amanile*) mixed altogether and asked to silently read them indicating only the real words, while continuously repeating aloud the syllable *la*. They were given 60 seconds to find as more words as possible: the final score was calculated by subtracting the number of errors from the total number of words correctly identified by the subject and transformed into z-scores.

We also assessed spelling, by administering a word dictation task, consisting of 56 real words, varying in length and frequency, dictated at a consistent rhythm of every 2 s (LCS-SUA). As for the scoring, we measured the number of spelling errors committed by participants and transformed them in z-scores.

Finally, we evaluated text comprehension by means of the *Prova di comprensione del testo (Brano A2)* taken from the Batteria MT Avanzate-3-Clinica (Cornoldi et al., 2017).

The LCS-SUA battery is widely recognized for its reliability and validity in assessing reading skills in Italian in adult individuals. The test–retest reliabilities reported, which were computed considering only small groups of typical readers, were good for word reading speed (r = 0.79), nonword reading speed (r = 0.76), and word spelling accuracy (r = 0.71), while they were lower for word reading accuracy (r = 0.54) and (r = 0.56). This might be due to the very low mean number of errors observed in typical readers (unfortunately, although all tests were normed for adults, the test–retest reliability data are not provided for dyslexics, as typically happens in Italian batteries; see Cornoldi et al. (2022) for a discussion on these aspects).

#### Linguistic measures

To gather a complete picture of the linguistic skills of adults with dyslexia, we added to our protocol a series of measures addressing their phonological and morphological skills, as well as their lexical access skills, which, as highlighted above, are key predictors of reading development.

Phonological competence was examined by means of four tasks: nonword repetition, spoonerisms, syllabic and phonemic inversion. As for nonword repetition, we administered the task developed by Vender et al. (2020), which comprised 40 stimuli of different lengths, ranging from two to five syllables, and complexity, encompassing CV, CVC, CCV, CCVC, CVVC structures (e.g. *buna*: two syllables, low complexity; *chestangutoldri*: five syllables, high complexity). Participants were asked to repeat the given nonwords, which were pre-recorded by a female native speaker of Italian; 1 point was given if the subjects repeated the whole stimulus correctly and 0 points in case of mistakes.

Regarding the Spoonerisms task, as in Vender and Melloni (2021), participants were given two two-syllable words (e.g., *cane* ‘dog’ and *rosa* ‘rose’) and instructed to exchange the initial sound of each word to form two distinct existing words (e.g., *rane* ‘frogs’ and *cosa* for ‘thing’). Two points were assigned if both words were accurately generated, 1 point if only one word was correctly produced, and 0 if no correct answers were provided. Response times were also measured. In Phonemic Inversion, participants were orally presented with a word, as *sapone* (‘soap’) and were asked to say one by one the phonemes composing the word in the reverse order (e.g. /e/-/n/-/o/-/p/-/a/-/s/). The stimuli ranged from two to three syllables; two syllable words comprised CV, CCV and CVC syllables, while three-syllable words only had CV syllables. One point was attributed if all phonemes were recalled correctly in the reverse order, 0 points for mistakes. Response times were also measured.

Similarly, in Syllabic Inversion, participants were orally presented with a word, like *pomodoro* (‘tomato’), and were asked to say aloud the syllables composing the word in the reverse order (*ro-do-mo-po*). Stimuli had two to four syllables and the structure was CV and CCV for two-syllables and only CV for three- and four-syllables. The scoring procedure was the same as in the preceding task. In both Phonemic and Syllabic Inversion tasks, we included only highly regular words with one-to-one mappings between graphemes and phonemes, with no orthographic complexities.

To address morphological competence, we analyzed their inflectional and derivational morphology skills using nonwords, inspired by the well-known Wug Test (Berko, 1958). As for inflection, we asked subjects to produce the plural of pseudo-nouns (e.g. *Questo è un* folo*, questi sono un po’ di*…target: foli; ‘This is a *fol-o*_MascSing_, these are some…target: *fol-i*_MascPlu_’) or the past participle of pseudo-verbs (e.g. *Qui Pippo si è messo a* pindare*. Cos’ha fatto?* Target*: Ha* pindato*;* ‘Here Goofy started to *pind-are*_Inf_*.* What has he done? Target*:* He has pind-ato_PastPart_). There were 15 items for Nonword Pluralization task and 9 items for the Past Participle Inflection task; 1 point was credited for correct inflections, 0 points for errors; phonological errors were not penalized (see Melloni and Vender (2022) for more details on these tasks). Both tasks were administered orally.

A Nonword Suffix Choice task, based on Nagy et al., (2006) and Piccinin & Dal Maso (2023) assessed derivational morphology. Participants were presented with a set of 10 written sentences with a missing word and were asked to complete a sentence with one out of four given nonwords (e.g. *Tutti ammiravano il maestro per la sua grande* ________. ‘Everyone admired the teacher for his great _________.’ Options: a) draposa; b) drapabile; c) drapezza; d) drapista; target: drapezza). The four options were nonwords obtained by a phonologically legal combination of an invented root and an existent suffix, in such a way that three of them could be ruled out based on syntactic and semantic properties of the suffix. In this example, *draposa* and *drapabile* can be excluded since they feature adjectival suffixes (-*osa* and -*bile*), whereas in this sentence a noun is required. *Drapista*, instead, can be excluded based on the semantics of the suffix -*ista*, which generates agentive nouns, while in this context we expect an abstract noun, which is compatible with the suffix -*ezza*. The nonword *drapezza* should then be selected. The task included 10 nonwords; 1 point was assigned to each correctly identified nonwords, 0 points otherwise.

Finally, we addressed lexical access by administering a rapid automatized naming task (RAN), in which participants were asked to rapidly name sequences of digits, letters, numbers and objects. For every set, there were 5 items repeated 5 times, for a total of 50 items each. One point was credited to each correctly named word; hesitations and self-corrections were not considered errors. Response times were also measured.

### Literacy intervention

The intervention was originally developed for this study based on what was reported as effective in previous interventions in adults and discussed above (see, in particular, the systematic review by Vender et al., 2022). It involved the independent use of a web application (on a computer, tablet, or smartphone) for three days a week (each session lasting approximately 15–20 min) for eight weeks. The developed activities were aimed at enhancing both reading, in terms of speed and accuracy, and spelling skills, and they were divided into different typologies.

The first activity, “Rapid Reading”, which involved rapid reading with modulation of on-screen persistence, was the most frequently used, characterizing half of the exercises proposed in each session. Participants were shown a stimulus (syllable, word, or nonword) which remained just for a limited time on the screen. As soon as the stimulus disappeared, they were asked to rewrite it in a designated space. Immediate feedback was provided, and in case of an error the correct word was shown. On-screen persistence was customized according to each participant's reading proficiency, as determined during the pre-assessment phase. This duration gradually decreased as participants advanced through levels, with progression to the next level contingent upon achieving an accuracy rate higher than 80% for each task. The duration of on-screen persistence ranged from 1200 to 100 ms, based on the length and complexity of the stimuli to be read. Each task was composed of stimuli of the same type, balanced for number of syllables, syllabic structure and frequency, which were presented with the same on-screen persistence: to exemplify, the first session comprised two-syllable words like *camera* ‘room’ shown for 600 ms, and the last comprised five-syllable words like *capitalismo* ‘capitalism’ displayed for 500 ms. The goal of this activity was to improve reading speed while maintaining correctness.

In “Pick the correct word”, participants were presented with two words, and they had to select the correctly spelled one (e.g. *filastrocca* vs. *vilastrocca, ‘nursery rhyme’). This exercise was proposed adopting either the written or the auditory modality. There were no time constraints and feedback was always provided.

In “Listen and choose the right match”, participants were presented with an auditory stimulus (word or nonword) and asked to select the correct written form from two alternatives; the wrong options were phonologically or visually similar to the correct one, in order to enhance discrimination between similar stimuli, or they contained spelling errors based on Italian orthographic conventions (e.g. real words: *chiarore*, *ciarore, ‘dim light’; nonwords: *bedimo* vs. *dedimo*, when the spoken form /be'dimo/ was given).

In the “Dictation” activity participants were presented with an auditory stimulus (either a word or a nonword) that they had to rewrite in a space below. Finally, in the “Lexical decision” task participants were presented with two stimuli, a correctly spelled word and a visually or phonologically similar nonword containing a spelling error (e.g. *imbianchino* vs. *indianchino*, ‘house painter’). The participant's task was to select the existing word.

For all the activities mentioned above, each exercise included 10 to 30 stimuli of the same type, based on various criteria, including number of syllables, syllabic and orthographic complexity, morphological structure, syntactic category, and lexical frequency. In terms of number of syllables, tasks were designed to feature items with consistent syllabic lengths, spanning from 2 to 5 syllables. The transition from 2-syllables to 5-syllables stimuli was progressive throughout the training, also in relation to syllabic and orthographic complexity. As for syllabic complexity, the initial tasks exclusively included simple syllables, while subsequent levels encompassed both simple and complex structures. Orthographic complexity was carefully managed by introducing difficult orthographic groups sequentially (e.g. “sce” in *scena* ['ʃɛ:na] ‘scene’ or “schie” in *schiena* [ˈskjɛna], ‘back’). This approach aimed to improve the automatization of decoding and writing skills. Subsequently, complex orthographic groups were presented in combination with others that shared similarities either phonologically or orthographically (e.g., gli/li, chi/ghi/che/ghe, schi/sci…), and that are notoriously difficult for dyslexics.

Starting from the 6th session, morphological analysis was introduced: alongside lists of simple words, participants were exposed to lists featuring complex derived words, stimulating an implicit morphological decomposition of the word to identify roots and affixes. Suffixes were gradually introduced based on their frequency, including -*zione*, -*mento*, -*mente*, -*tore*/-*trice*, -*bile*, -*ale*, -*oso*, -*orio*, -*esco*, -*ezza*, -*ismo*, -*anza*, -*enza*, -*aggio*, -*tura*, -*ificare*, -*eria*. The prefixes *super*- and *iper*- were also presented.

Stimuli were divided based on their syntactic category; given their prevalence in the Italian lexicon (60.7%), most stimuli were nouns, followed by verbs and adjectives. Lexical frequency was also controlled: words were exclusively drawn from the *Nuovo Vocabolario di Base della Lingua Italiana* (De Mauro & Chiari, 2016), encompassing the 7000 most frequently used Italian words. We decided to focus on frequent words since they are more prevalent in non-specialized written texts and thus more useful for our purposes. Importantly, all trained items appeared only once throughout the training and none of the stimuli was the same of those used in the pre- and post-intervention tasks.

### Procedure

All participants were first individually administered the literacy and language tasks described above. As the task administration started during the Covid-19 pandemic, in 2021, all tests had to be administered online via zoom. Participants were asked to connect using their laptop; the experimenter shared their screen, making sure that the subjects were properly visualizing it and correctly seeing all displayed stimuli. Each session was recorded. All tests were coded twice by two blind experimenters, who were not informed about the purposes of the whole protocol; the inter-rater reliability was higher than 0.90 for all the tasks and the few disagreements were resolved after a discussion between the coders. The pre-intervention assessment was divided into sessions of approximately 45–60 min each, administered within one week. The order of the tasks administered was as follows: in the first session, we administered Text Reading, Word Reading, Nonword Reading, Lexical Decision under Articulatory Suppression, Nonword Repetition, Spoonerisms, and SPM Raven. In the second session, we assessed Text Comprehension, Syllabic Inversion, Phonemic Inversion, Nonword Pluralization, Past Participle Inflection, Nonword Suffix Choice, RAN and Spelling.

Dyslexics were then randomly divided into an experimental group and a control group, based on their order of enrollment. The first 24 participants were then assigned to the experimental group and administered the intervention, which lasted 8 weeks, as discussed above, while the participants who enrolled later were assigned to the control group, consisting of 20 individuals, who did not receive any training. Four subjects of the experimental group and 2 of the control group dropped out before the end of the whole protocol, resulting in final sample sizes of 20 participants for the experimental group and 18 for the control group.

Within one week after the end of the training (and within 9 weeks for the control group), all participants were administered all the reading measures administered in the pre-intervention, including Phonemic and Syllabic Inversion. It should be emphasized that none of the stimuli addressed in the pre- and post-assessment were part of the items trained during the intervention.

Several steps were also pursued to ensure intervention fidelity. First, all participants were given detailed instructions on the procedures to be followed during each session, explaining that they had to complete the activities assigned within three days. As explained above, all activities were delivered using a web app, which provided a uniform interface and controlled the timing and presentation of tasks, thus ensuring consistent delivery. Participants were also instructed on how to contact the experimenter in case of issues with the technological app, in order to ensure immediate assistance. Moreover, the experimenter constantly monitored their activity and engagement, ensuring they had no difficulties accessing the app and completing the assigned activities. Detailed logs recording the activities completed, their duration, and the participants’ accuracy were collected daily and monitored by the experimenter, who constantly supervised the subjects’ activity, contacting them if they did not complete the assigned tasks and evaluating their progress and their accuracy to assign the next activities.

The activities assigned to each participant were designed to be similar, to ensure uniformity of treatment, but they were also adapted based on each participant's progress and reading rate. For instance, the persistence of the stimuli on the screen in the “Rapid Reading” activity was determined individually based on the participants’ reading rates as assessed in the pre-assessment phase, and it was adapted based on the participants’ progress (increased, if accuracy in reading across the activities proposed in each session was above 80%, or decreased if it was lower).

This approach allowed for personalization within a consistent intervention structure, ensuring that the tasks were appropriately challenging and supportive of each individual's needs. Finally, regular feedback was collected from the participants to identify potential issues; no participants showed difficulties in accessing the app and completing the assigned activities in due time.

The study was approved by the local ethics committee and conducted in accordance with the standards specified in the 2013 Declaration of Helsinki; written informed consent was collected from each participant.

## Results

### Literacy and linguistic skills of typical and atypical readers

Table 1 summarizes means and SDs of the two groups in the literacy measures administered, indicating that dyslexics underperformed controls in all measures.

**Table 1:** Means (SDs) of the two groups in the reading tasks, spelling and text comprehension

Group	Text Reading (composite z-score)	Word Reading (composite z-score)	Nonword Reading (composite z-score)	Lexical Decision (z-score)	Spelling (z-score)	Text Comprehension (z-score)
**Controls**	-0.86 (1.17) [Min: -14.43; Max: -0.05]	-0.27 (0.70) [Min: -2.58; Max: 0.97]	-0.34 (0.83) [Min: -2.57; Max: 2.57]	-1.22 (0.83) [Min: -2.68; Max: 1.00]	-0.95 (1.55) [Min: -4.98; Max: 0.77]	0.70 (0.78) [Min: -0.38; Max: 1.88]
**Dyslexics**	-3.98 (2.66) [Min: -3.44; Max: 0.72]	-2.71 (1.90) [Min: -8.35; Max: -0.39]	-2.50 (1.65) [Min: -7.20; Max: -0.22]	-2.66 (1.06) [Min: -5.25; Max: -0.67]	-2.84 (2.90) [Min: -0.73; Max: 0.77]	-0.10 (1.04) [Min: -2.83; Max: 1.88]

To verify the presence of significant group differences, we fitted a series of linear models to predict each variable with Group (controls vs. dyslexic) as fixed factor. Dyslexics performed significantly more poorly than controls in Text Reading (β = -3.12, t(86) = -7.12, p < 0.0001; Cohen’s d = 1.52), Word Reading (β = -2.44, t(86) = -7.99, p < 0.001; Cohen’s d = 1.70), Nonword Reading (β = -2.16, t(86) = -7.79, p < 0.001; Cohen’s d = 1.66), Lexical Decision under Articulatory Suppression (β = -1.44, t(86) = -7.11, p < 0.001; Cohen’s d = 1.52), spelling (β = -1.90, t(74) = -3.46, p < 0.001; Cohen’s d = 0.80) and Text Comprehension (β = -0.79, t(85) = -4.01, p < 0.001; Cohen’s d = 0.86).

Results of the phonological tasks are reported in Table 2; as can be observed, dyslexics were less accurate in all tasks, showing also markedly longer response times.

**Table 2:** Mean accuracy (SDs) of the two groups in the phonological tasks

	Nonword Repetition (Accuracy rate)	Spoonerisms (Accuracy rate)	Spoonerisms (Time, in seconds)	Syllabic Inversion (Accuracy rate)	Syllabic Inversion (Time, in seconds)	Phonemic Inversion (Score)	Phonemic Inversion (Time, in seconds)
**Controls**	0.84 (0.09) [Min: 0.63; Max: 1.00]	0.91 (0.09) [Min: 0.55; Max: 1.00]	63.89 (45.08) [Min: 24; Max: 273]	0.96 (0.11) [Min: 0.27; Max: 1.00]	72.23 (22.49) [Min: 37; Max: 146]	0.96 (0.06) [Min: 0.80; Max: 1.00]	88.16 (31.13) [Min: 34; Max: 193]
**Dyslexics**	0.71 (0.13) [Min: 0.40; Max: 1.00]	0.82 (0.15) [Min: 0.35; Max: 1.00]	137.02 (76.36) [Min: 44; Max: 499]	0.90 (0.12) [Min: 0.47; Max: 1.00]	114.00 (39.44) [Min: 50; Max: 230]	0.90 (0.10) [Min: 0.60; Max: 1.00]	129.81 (41.43) [Min: 60; Max: 263]

The statistical analysis confirmed that dyslexics underperformed controls in all measures, being less accurate in Nonword Repetition (β = -0.13, t(86) = -5.59, p < 0.001, Cohen’s d = 1.19), and both slower and less accurate in the other tasks, including Spoonerisms (accuracy: β = -0.09, t(86) = -3.62, p < 0.001, Cohen’s d = 0.77; time: β = 73.14, t(86) = 5.47, p < 0.001, Cohen’s d = 1.17), Syllabic Inversion (accuracy: β = -0.06, t(86) = -2.57, p < 0.05, Cohen’s d = 0.55; time: β = 41.77, t(85) = 6.05, p < 0.001, Cohen’s d = 1.30) and Phonemic Inversion (accuracy: β = -0.06, t(86) = -3.33, p < 0.01, Cohen’s d = 0.71; time: β = 41.66, t(86) = 5.33, p < 0.001, Cohen’s d = 1.14).

Results regarding morphological competence are instead shown in Table 3.

**Table 3:** Mean accuracy rates (SDs) of the two groups in the morphological tasks

Group	Nonword Pluralization	Past Participle Inflection	Nonword Suffix Choice
**Controls**	0.80 (0.11) [Min: 0.53; Max: 1.00]	0.83 (0.18) [Min: 0.33; Max: 1.00]	0.94 (0.08) [Min: 0.67; Max: 1.00]
**Dyslexics**	0.81 (0.14) [Min: 0.47; Max: 1.00]	0.71 (0.20) [Min: 0.33; Max: 1.00]	0.73 (0.18) [Min: 0.17; Max: 1.00]

Dyslexics performed similarly to controls in Nonword Pluralization (β = 0.01, t(86) = 0.40, p = 0.689, Cohen’s d = 0.09), whereas they showed lower accuracy in both Past Participle Inflection (β = -0.13, t(86) = -3.11, p < 0.01, Cohen’s d = 0.66) and Nonword Suffix Choice (β = -2.45, t(86) = -6.81, p < 0.001, Cohen’s d = 1.45).

Finally, accuracy and response times in rapid naming are reported in Table 4.

**Table 4:** Mean accuracy (SDs) and times (in seconds) of the two groups in the four RAN subtests

Group	RAN digits (accuracy rate)	RAN digits (time)	RAN letters (accuracy rate)	RAN letters (time)	RAN colors (accuracy rate)	RAN colors (time)	RAN objects (accuracy rate)	RAN objects (time)
**Controls**	1.00 (0.01) [Min: 0.96; Max: 1.00]	19.32 (3.04) [Min:14; Max: 26]	1.00 (0.01) [Min: 0.96; Max: 1.00]	21.21 (3.83) [Min: 15; Max: 33]	1.00 (0.01) [Min: 0.96; Max: 1.00]	27.47 (4.68) [Min: 22; Max: 41]	0.99 (0.01) [Min: 0.94; Max: 1.00]	28.00 (4.75) [Min: 21; Max: 41]
**Dyslexics**	1.00 (0.02) [Min: 0.92; Max: 1.00]	25.30 (5.90) [Min: 17; Max: 49]	0.97 (0.05) [Min: 0.78; Max: 1.00]	29.30 (6.80) [Min: 18; Max: 57]	1.00 (0.02) [Min: 0.90; Max: 1.00]	35.39 (6.52) [Min: 25; Max: 58]	1.00 (0.01) [Min: 0.96; Max: 1.00]	35.25 (5.83) [Min: 26; Max: 57]

As can be observed, accuracy was always at ceiling for both groups; only in letters dyslexics underperformed controls (β = -0.02, t(86) = -3.01, p < 0.01, Cohen’s d = 0.64); no differences were observed in the other subtests (digits: β = -0.00, t(86) = -1.42, p = 0.159, Cohen’s d = 0.30; colors: β = -0.00, t(85) = -0.12, p = 0.906, Cohen’s d = 0.25; objects: β = 0.00, t(85) = 1.13, p = 0.262, Cohen’s d = 0.24). Dyslexics were slower than controls in all sets: letters (β = 8.09, t(86) = 6.88, p < 0.001, Cohen’s d = 1.47), digits (β = 5.98, t(86) = 5.98, p < 0.001, Cohen’s d = 1.27), colors (β = 7.92, t(85) = 6.50, p < 0.001, Cohen’s d = 1.39) and objects (β = 7.25, t(85) = 6.35, p < 0.001, Cohen’ d = 1.36).

To summarize, dyslexics showed marked deficits in all literacy and language tasks administered.

### Evaluation of the intervention on adult dyslexics

To assess the effectiveness of the intervention, which was administered only to the dyslexics assigned to the experimental group, we compared their performance to that of the dyslexic control group in the literacy measures and in Phonemic and Syllabic Inversion tasks administered before and after the intervention. (see Table 5).

**Table 5:** Summary of results in the reading and spelling tasks across control and experimental groups, measured in pre- and post-intervention

Task	Group	Time of administration	Mean (SD)	Group comparisons at pre-test	Intervention effect
**Text Reading**	Control	t1 (pre-test)	-3.50 (2.58) [Min: -8.40; Max: -0.05]	p = .320	Group*Time: p < .01**
		t2 (post-test)	-2.91 (2.12) [Min: -7.7; Max: 0.01]		
	Experimental	t1 (pre-test)	-4.41 (2.94) [Min: -14.43; Max: -1.40]		
		t2 (post-test)	-2.20 (1.67) [Min: -6.84; Max: -0.45]		
**Word Reading**	Control	t1 (pre-test)	-2.47 (1.69) [Min: -6.15; Max: -0.39]	p = .510	Group*Time: p < .01**
		t2 (post-test)	-2.01 (1.23) [Min: -4.31; Max: -0.45]		
	Experimental	t1 (pre-test)	-2.87 (2.02) [Min: -8.35; Max: -0.8]		
		t2 (post-test)	-1.67 (1.45) [Min: -4.77; Max: -0.04]		
**Nonword Reading**	Control	t1 (pre-test)	-2.16 (1.55) [Min: -6.05; Max: -0.22]	p = .167	Group*Time: p = .075
		t2 (post-test)	-1.77 (1.00) [Min: -4.17; Max: -0.44]		
	Experimental	t1 (pre-test)	-2.95 (1.87) [Min: -7.2; Max: -0.50]		
		t2 (post-test)	-1.97 (1.50) [Min: -5.94; Max: 0.07]		
**Lexical Decision**	Control	t1 (pre-test)	-2.58 (1.08) [Min: -5.25; Max: -1.12]	p = .836	Group*Time: p = .891
		t2 (post-test)	-2.14 (1.07) [Min: -3.68; Max: -0.56]		
	Experimental	t1 (pre-test)	-2.51 (0.99) [Min: -4.35; Max; -0.67]		
		t2 (post-test)	-2.13 (1.06) [Min: -4.24; Max: -0.11]		
**Spelling**	Control	t1 (pre-test)	-1.39 (2.17) [Min: -7.19; Max: 0.77]	p < .01**	Group*Time: p < .001***
		t2 (post-test)	-0.98 (1.39) [Min: -4.53; Max: 0.77]		
	Experimental	t1 (pre-test)	-3.96 (3.22) [Min: -10.73; Max: 0.77]		
		t2 (post-test)	-0.98 (2.67) [Min: -8.07; Max: 0.77]		
**Reading Comprehension**	Control	t1 (pre-test)	-0.36 (1.11) [Min: -2.83; Max: 1.36]	p = .206	Group*Time: p = 0.908
		t2 (post-test)	-0.10 (1.14) [Min: -1.79; Max: 1.36]		
	Experimental	t1 (pre-test)	0.09 (1.07) [Min: -2.31; Max: 1.88]		
		t2 (post-test)	0.48 (1.02) [Min: -1.26; Max: 1.88]		

Group comparisons in pre-test assessments were calculated fitting a series of linear models on T1 (pre-test) data to predict each variable with Group (Experimental vs. Control). Intervention effects are indicated by the Group*Time interaction p-values

To assess the effectiveness of the intervention, we fitted a series linear mixed model (estimated using REML and nloptwrap optimizer) to predict each reading variable with Group (Experimental vs. Control) and Time (pre-test vs. post-test assessment), including Participant as a random effect. The participants’ age was also added as a covariate to check for possible effects on the intervention’s effects related to this aspect. Standardized parameters were obtained by fitting the model on a standardized version of the dataset. 95% Confidence Intervals (CIs) and p-values were computed using a Wald t-distribution approximation.

**Fig. 1:**
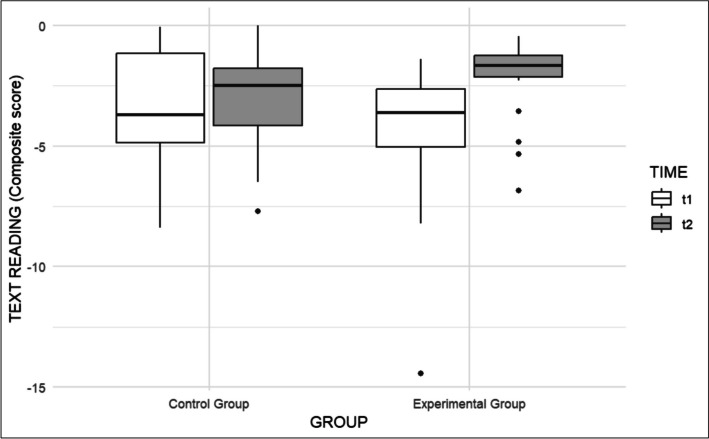
Comparison of pre- and post-test in Text Reading of the two groups before (t1) and after (t2) the intervention

As for Text Reading, we found no significant Group effect (β = -1.05, t(45.97) = -1.39, p = 0.171; Cohen's d = 0.45), Age (β = 0.19, t(35) = 1.80, p = 0.08; Cohen's d = 0.08), nor Time effect (β = 0.58, t(36) = 1.43, p = 0.16; Cohen's d = 0.25), but we found a statistically significant Group × Time interaction, indicating a higher performance in the post-test only for the experimental group, as displayed in Fig. 1 (β = 1.62, t(36) = 2.88, p < 0.01; Cohen's d = 0.70).

**Fig. 2:**
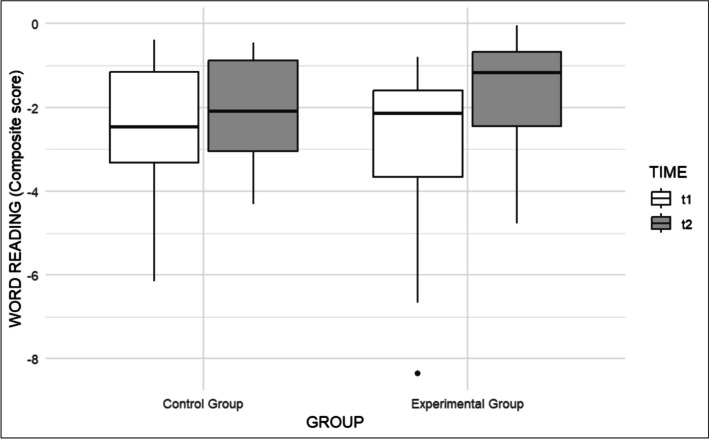
Comparison of pre- and post-test in Word Reading of the two groups before (t1) and after (t2) the intervention

Similarly, considering Word Reading, Group was non-significant (β = -0.51, t(40.09) = -0.99, p = 0.326; Cohen's d = 0.32), as well as Age, which only approached significance, suggesting that older participants tended to have a better reading performance (β = 0.14, t(35) = 1.92, p = 0.063; Cohen's d = 0.09). Time was significant (β = 0.46, t(36) = 2.37, p < 0.05; Cohen's d = 0.29) as well as the Group × Time interaction (β = 0.74, t(36) = 2.74, p < 0.01, Cohen's d = 0.46), indicating that the experimental group showed higher improvement than the control group (see Fig. 2).

**Fig. 3:**
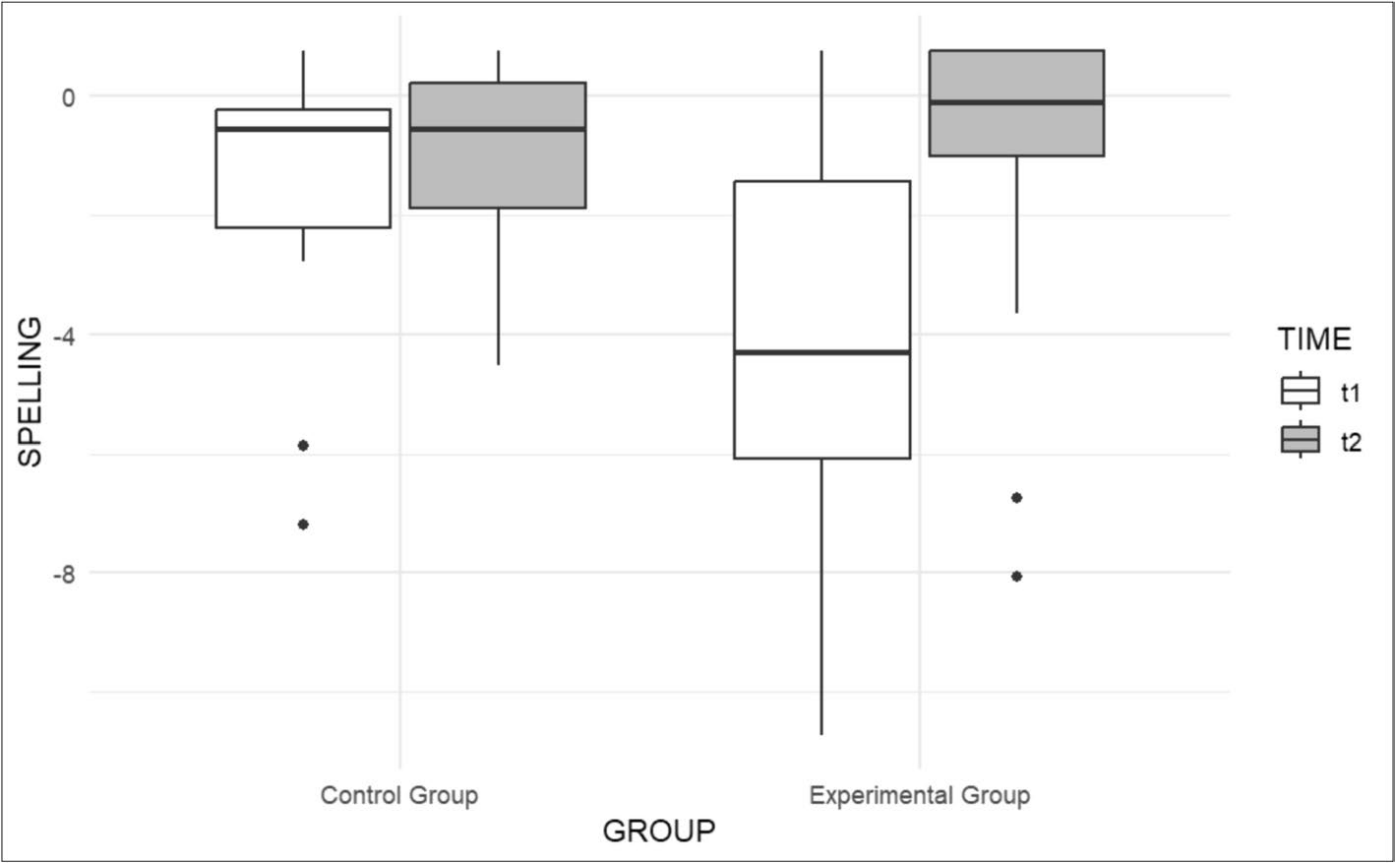
Comparison of pre- and post-test in Spelling of the two groups before (t1) and after (t2) the intervention

As for Nonword Reading, we found instead no effects of Group (β = -0.86, t(43.61) = -1.77, p = 0.084; Cohen's d = 0.57), Age (β = 0.10, t(35) = 1.47, p = 0.150; Cohen's d = 0.07) or Time (β = 0.39, t(36) = 1.65, p = 0.108; Cohen's d = 0.260), while Group × Time only approached significance, with a marginal positive effect of treatment for dyslexics (β = 0.59, t(36) = 1.81, p = 0.079; Cohen's d = 0.392). In Lexical Decision we found that both groups had a higher performance in the post-test, with no other significant differences (Group: β = 0.06, t(46.05) = 0.185, p = 0.854; Cohen's d = 0.06; Age: β = 0.08, t(35.23) = 0.172, p = 0.864; Cohen’s d = 0.01; Time: β = 0.44, t(35.19) = 2.33, p < 0.05; Cohen's d = 0.41; Group × Time: β = -0.04, t(35.40) = -0.14, p = 0.891; Cohen's d = 0.03). No significant differences were observed in Text Comprehension (Group: β = 0.41, t(44.71) = 1.152, p = 0.256; Cohen's d = 0.38; Age: β = 0.06, t(34.27) = 1.13, p = 0.267; Cohen's d = 0.05; Time: β = 0.25, t(34.23) = 1.339, p = 0.189; Cohen's d = 0.23; Group × Time (β = 0.03, t(34.42) = 0.10, p = 0.918; Cohen's d = 0.03). Conversely, in Spelling we found that the experimental group was less accurate than the control group (β = -2.52, t(45.35) = -3.09, p < 0.01; Cohen's d = 1.01), while there were no effects of Age (β = 0.07, t(34.91) = 0.60, p = 0.55; Cohen's d = 0.03) or Time (β = 0.41, t(33.06) = 0.91, p = 0.367; Cohen's d = 0.16) but a significant Group × Time interaction, indicating that improvements were significant for the experimental group, as displayed in Fig. 3 (β = 2.40, t(33.70) = 3.77, p < 0.001; Cohen's d = 0.96).

To verify the presence of improvements in phonological awareness, we considered both time and accuracy in Phonemic Inversion and Syllabic Inversion. Mean results are reported in Table 6.

**Table 6:** Summary of the accuracy (percentage) and time (seconds) for phonemic and syllabic inversion tasks across control and experimental groups, measured in pre- and post-intervention

Task	Group	Time of administration	Mean (SD)	Group comparisons at pre-test	Intervention effect
**Phonemic inversion** **(Accuracy, in percentage)**	Control	t1 (pre-test)	0.95 (0.06) [Min: 0.80; Max: 1.00]	p < .01 **	Group*Time: p = .108
		t2 (post-test)	0.95 (0.06) [Min: 0.80; Max: 1.00]		
	Experimental	t1 (pre-test)	0.86 (0.10) [Min: 0.60; Max: 1.00]		
		t2 (post-test)	0.91 (0.14) [Min: 0.53; Max: 1.00]		
**Phonemic inversion** **(Time, in seconds)**	Control	t1 (pre-test)	115.29 (37.72) [Min: 60; Max: 204]	p = .070	Group*Time: p < .01**
		t2 (post-test)	106.11 (50.86) [Min: 57; Max: 285]		
	Experimental	t1 (pre-test)	139.75 (41.34) [Min: 89; Max: 263]		
		t2 (post-test)	99.79 (27.30) [Min: 69; Max: 166]		
**Syllabic inversion (Accuracy, in percentage)**	Control	t1 (pre-test)	0.95 (0.08) [Min: 0.80; Max: 1.00]	p = .05	Group*Time: p = .081
		t2 (post-test)	0.92 (0.10) [Min: 0.73; Max: 1.00]		
	Experimental	t1 (pre-test)	0.86 (0.14) [Min: 0.47; Max: 1.00]		
		t2 (post-test)	0.91 (0.10) [Min: 0.67; Max: 1.00]		
**Syllabic inversion (Time, in seconds)**	Control	t1 (pre-test)	103.22 (38.65) [Min: 50; Max: 197]	p = .139	Group*Time: p = 0.212
		t2 (post-test)	81.56 (22.83) [Min: 42; Max: 128]		
	Experimental	t1 (pre-test)	123.10 (42.05) [Min: 66; Max: 230]		
		t2 (post-test)	84.58 (27.08) [Min: 56; Max: 153]		

Group comparisons in pre-test assessments were calculated fitting a series of linear models on T1 (pre-test) data to predict each variable with Group (Experimental vs. Control). Intervention effects are indicated by the Group*Time interaction p-values

As for Phonemic Inversion, the experimental group underperformed the control group in accuracy, while no other effects were found (Group: β = -0.10, t(60.86) = -2.96, p < 0.01; Cohen’s d = 0.97; Age: β = -0.01, t(35.28) = -0.39, p = 0.70; Cohen’s d = 0.02; Time: β = -0.01, t(35.33) = -0.14, p = 0.886; Cohen’s d = 0.04; Group × Time: β = 0.06, t(35.70) = 1.63, p = 0.111; Cohen’s d = 0.60). In response time, we found instead a marginally slower performance of the experimental group (Group: β = 25.55, t(47.22) = 1.97, p = 0.054; Cohen’s d = 0.648), with no effects of Age (Group: β = -2.510, t(35.75) = -1.42, p = 0.165; Cohen’s d = 0.06), Time (β = -10.00, t(34.98) = -1.37, p = 0.179; Cohen’s d = 0.25) but a significant Group × Time interaction, showing that performance improved significantly for the experimental group (β = -29.69, t(34.95) = -2.95, p < 0.01; Cohen’s d = 0.75).

As for Syllabic Inversion, we found a Group effect in accuracy, with the experimental group performing more poorly than the control group (β = -0.09, t(30.48) = -2.64, p < 0.05; Cohen’s d = 0.86), but no effects of Age (β = 0.00, t(29.14) = 0.36, p = 0.718; Cohen’s d = 0.02), Time (β = -0.03, t(29.15) = -1.11, p = 0.275; Cohen’s d = 0.27), while Group × Time only approached significant (β 0.07, t(29.48) = 1.76, p = 0.088; Cohen’s d = 0.60). In response times, instead, we found a significant effect of Group (β = 22.25, t(53.83) = 2.06, p < 0.05, Cohen’s d = 0.67), Time (β = -21.67, t(31.78) = -2.73, p < 0.05, Cohen’s d = 0.66) and Age (β = -3.12, t(31.78) = -2.25, p < 0.05, Cohen’s d = 0.09), but no Group × Time interaction (β = -13.80, t(32.10) = -1.25, p = 0.219, Cohen’s d = 0.42), indicating that the experimental group was generally slower, that both groups were faster in the post-test and that younger participants performed better, but that no effects of the intervention were observed.

To summarize, the intervention produced significant gains in text reading, word reading, spelling, phonemic inversion (response times), and only marginally in nonword reading and syllabic inversion (response times), independently of the participants’ age. No effects were observed instead in lexical decision and text comprehension.

## Discussion

This study aimed to investigate the still unexplored domain of young adults with dyslexia, analyzing their literacy and linguistic profile and assessing the efficacy of an intervention designed to improve their reading and spelling skills. Since gaining a more complete understanding of the linguistic and cognitive profiles of adults with dyslexia is crucial for designing targeted interventions and support strategies, we started by providing an in-depth assessment of their ability in their decoding, fluency, comprehension, and writing skills, alongside an analysis of their phonological, morphological and lexical access skills, which, as emphasized above, are reported in the literature as key predictors of reading proficiency in both children and adults (Carioti et al., 2021; Snowling et al., 2020). To do so, we compared a group of 44 young adults with a diagnosis of dyslexia, whose age was comprised between 16 and 30 years old, with a group of 44 age-matched typical readers.

Results showed that dyslexics performed more poorly than controls across all literacy measures: reading difficulties were marked in all tasks administered, which varied in the nature of the stimuli targeted and the complexity of the task; specifically, dyslexics struggled in reading lists of isolated words and nonwords, as well as in reading connected text, where the coordination of word reading fluency with higher-level, context-driven processing is required to access the overall content conveyed by the text (Wallot et al., 2013). Furthermore, deficiencies were observed in nonword reading too, suggesting impairments in the functioning of both the sublexical route (in reading nonwords) and the lexical route (in reading words and text; Coltheart et al., 2001). Difficulties were also conspicuous in spelling and in lexical decision under articulatory suppression, a more complex task in which participants were asked to select only real words from a list of words and nonwords while uttering the syllable *la*. Text comprehension was also impaired, confirming that dyslexic adults’ enduring difficulties in decoding have negative effects on the interpretation of written materials, too. Results thus highlight that literacy difficulties are severe in adults with dyslexia as well.

Regarding our second research aim, we also observed the presence of severe phonological difficulties in dyslexics, in all measures administered, from the simplest as nonword repetition, to the more complex requiring higher working memory resources, like spoonerisms and phonemic and syllabic inversions. In these last three measures, dyslexics were both less accurate and markedly slower than controls, thus confirming the persistent nature of phonological awareness deficits in dyslexia and the difficulty in singling out and manipulating phonemes and syllables (Bruck, 1992; Reis et al., 2020), even in a language with a transparent writing system like Italian. RAN was also impaired across all measures, with dyslexics being slower than typical readers in naming letters, digits, objects and colors and also less accurate in naming letters, confirming the difficulties in lexical access commonly reported in dyslexia (Carioti et al., 2021; Nergård-Nilssen & Hulme, 2014; Vukovic et al., 2004). Deficits also emerged in morphological competence: while dyslexics reached the same accuracy as controls in pluralizing invented words, impairments remained in verbal inflectional morphology, in the production of past participles of pseudowords, and in derivational morphology. While it has been proposed that dyslexics can resort to their morphological abilities to compensate for their phonological difficulties, the morphological awareness of adult dyslexics has been scarcely studied so far (Bowers et al., 2010; Elbro & Arnbak, 1996; Goodwin & Ahn, 2013). Our results seems to contradict those from Cavalli et al. (2017) and Martin et al. (2014), who reported similar performance of adults with and without dyslexia in morphological tasks; notice however that in these studies participants were exposed to real words, while in our research they were required to manipulate nonwords. This suggests that lexical competence may have played a role in their studies, and that morphological difficulties still arise when they were addressed more directly through tasks involving nonwords. To sum up, the difficulties displayed by dyslexic adults in our morphological tasks are similar to those observed in children, confirming that these deficits persist into adulthood (Swanson & Hsieh, 2009).

Our third research objective was to determine whether adults could enhance their reading and spelling abilities through targeted linguistic training. To address this inquiry, we devised an innovative training program consisting of 24 sessions, each lasting 15–20 minutes which was delivered via a web application, totaling 8 hours of personalized instruction. Activities were designed to speed up decoding, automatizing grapheme-phoneme conversion rules and encouraging internal analysis of stimuli by identifying syllables, complex orthographic clusters, and morphemes, capitalizing on the crucial role played by phonological and morphological awareness in reading development. Drawing on what emerged as effective in the literature on adults with dyslexia (Vender et al., 2022), our training had a multimodal nature, stimulating both phonological and morphological processing, and also enhancing fluency through a computerized design aimed to speed up decoding. Moreover, both reading and spelling were addressed in the activities that we developed, following Hall et al. (2023), who reported the presence of reading instruction alongside spelling instruction as maximally effective. Importantly, all activities were personalized, selected based on the participants' literacy skills, as they were assessed before the intervention, as well as on their performance throughout the training sessions; moreover, stimuli were carefully chosen and balanced considering lexical frequency, orthographic complexity, and syllabic complexity to specifically address challenges faced by dyslexics, including difficulties in discriminating phonologically or visually similar items.

Results of pre- and post-intervention assessments confirmed the efficacy of the training, which was particularly evident in the enhanced proficiency in word and text reading as observed in the experimental group with respect to the control group. Notably, our intervention demonstrated a positive impact on spelling as well, which was less frequently explored in previous research. These findings thus align with existing studies that underscore the potential for literacy skill enhancement in adults (Bar-Kochva, 2016; Horowitz-Kraus, 2016; Shiran & Breznitz, 2011; Vender et al., 2022).

Only marginal training effects were observed in nonword reading, while no effects were found in lexical decision under articulatory suppression. We can attribute these results, respectively, on the one side to the fact that we focused more on real words than on nonwords in our training, and on the other side to the consideration that these tasks require more complex skills, involving working memory besides decoding, which were not specifically targeted by our activities. Similarly, given that text comprehension requires a complex interplay of cognitive and linguistic processes, involving both lower-order and higher-order cognitive skills (Grabe & Yamashita, 2022), the absence of substantial effects on text comprehension, consistent with existing research (Vender et al., 2022), suggests that enhancing this ability demands more comprehensive training than simply focusing on decoding proficiency.

Besides word and text reading and spelling, our intervention had a significant effect on the speed of phonological processing as well, with the experimental group exhibiting significantly faster response times in phonemic inversion tasks post-intervention compared to the control group; a trend for significance was observed also for response times in syllabic inversion. Although dyslexics of the experimental groups were slower than those of the control group, the gap was significantly reduced in the post-test after the intervention, signaling a significant improvement attributable to the training. This aligns with previous research indicating that boosting literacy skills can enhance phonological skills, too (Eden et al., 2004; Shiran & Breznitz, 2011). The intervention's specific focus on the phonemic structure of the stimuli, specifically training the discrimination between phonologically or visually similar phonemes, likely contributed to the higher improvements in phonemic inversion than in syllabic inversion.

Summarizing, our findings offer compelling evidence that literacy skills in adults can be effectively enhanced through targeted interventions. We believe that the personalized nature of our training program, which was tailored to each participant's specific cognitive and linguistic profile, played a pivotal role: personalization is indeed a key feature, especially important for adults, whose literacy skills can exhibit substantial heterogeneity. The individualized administration of the training was thus arguably beneficial, aligning with previous research that supports the efficacy of one-on-one instruction for people with diverse literacy profiles (Galuschka et al., 2020). Finally, we believe that the incorporation of a computerized training based on the participants’ reading rate, mirroring the reading acceleration training (Breznitz et al., 2013; Horowitz-Kraus, 2016) and imposing time constraints on reading, is crucial in promoting fluency. This finding reinforces the notion that fluent reading hinges, at least partially, on rapid information processing, and that this skill exhibits sufficient plasticity and adaptability to be effectively trained, even in adults and in individuals with dyslexia (Breznitz et al., 2013; Horowitz-Kraus, 2016).

This study has important educational implications. First, it underscores the persistent nature of reading and spelling deficits in adults with dyslexia, thus highlighting their need for ongoing support and targeted literacy interventions beyond childhood and adolescence. In addition, our results show that significant improvements can be obtained by integrating training of phonological and morphological skills with computerized activities aiming at automatizing and speeding up decoding. These skills should then be addressed to foster reading development, not only in specific intervention programs delivered by practitioners, but also within everyday classroom activities by teachers and educators. Teachers can indeed incorporate phonological and morphological exercises into regular classroom instruction in an inclusive manner, benefiting both typical and atypical readers, thus ensuring that all students, including poor readers who are not diagnosed as dyslexic, receive the necessary support to develop strong reading skills.

## Conclusions, limitations and indications for future research

Although dyslexia is a lifelong condition, research on adults is still very sparse, and mostly focuses on English, a language with an opaque orthographic system. Our study aimed to fill this gap by contributing novel insights, not only expanding the understanding of the literacy and linguistic profile of adults with dyslexia in Italian, a transparent orthography that has been underinvestigated so far, but also assessing the effectiveness of an intervention aimed at enhancing reading and spelling skills in adulthood.

Our findings revealed that dyslexic adults continue to face significant challenges in reading, across different materials and conditions, in text comprehension, and in spelling, even within a transparent writing system. Additionally, they exhibit impairments in phonological and morphological processing, as well as in rapid naming. These findings underscore the persistent nature of dyslexia's associated deficits, encompassing not only literacy but also linguistic abilities, thus highlighting the importance of comprehensive assessments, including literacy and language skills, for both children and adults, to gain a deeper understanding of dyslexia's trajectory from childhood to adulthood.

Importantly, our study also demonstrated the feasibility of interventions in adults to enhance literacy skills through targeted training, as our personalized computerized training program yielded significant improvements in text and word reading, spelling, and phonological processing speed. This critical finding challenges the prevailing notion that interventions are ineffective for adults with dyslexia, often leading them to resign to their literacy difficulties. Instead, adults with dyslexia deserve the opportunity to enhance their reading skills, and thus to pursue not only higher education but also cultural, social and professional fulfillment.

While we believe that our study offers a significant contribution to the existing literature, we acknowledge as a potential limitation the absence of a follow-up assessment to verify the persistence of training effects. Future research should address this aspect, recognizing the potential benefits of continued practice, particularly in reading fluency, to maintain and further enhance acquired reading skills beyond the intervention period. Another limitation is the study’s reliance on online testing due to the Covid-19 pandemic, which, despite the rigorous control of data collection and the careful monitoring of progression in the intervention, might have introduced variability in test administration as well as in the participants’ engagement. Future research could address these limitations by including a larger sample size and ensuring in-person assessment to enhance the generalizability and robustness of the findings. Moreover, while the assessments used in this study were normed for adults, it is important to note that they did not report reliability data. While we do not believe that this impacts the validity of the results achieved in our study, confirming the reliability of these measures specifically for adults with dyslexia highlights an area for further research. In addition, despite randomization based on the order of enrollment due to technical constraints of our study, we could not carefully match participants across intervention and control groups, which may introduce bias and affect the comparability of the groups. The use of a passive control group, which did not receive any intervention, constitutes another possible limitation. Future studies should thus employ active control groups and more careful randomization procedures to enhance the robustness and generalizability of these findings.

## Data Availability

The data that support the findings of this study are available on request from the corresponding author.

## Footnotes

1 Language deficits are typically observed also in children with Developmental Language Disorder (DLD; former: Specific Language Impairment), a disorder that is characterized by marked difficulties in receptive and expressive competence which tend to co-occur with dyslexia (Bishop & Snowling, 2004; Leonard, 2014). It has been observed that some children with dyslexia meet the diagnostic criteria for DLD, but the nature of the overlap between dyslexia and DLD is not clear yet (Snowling et al., 2020).
